# Correspondence

**Published:** 1869-07

**Authors:** Frank Abbott

**Affiliations:** Dean of the Faculty, N.Y. C. D., New York


### Correspondence.

---

NEW YORK, July 12, 1869.

EDS. DENTAL REGISTER—*Dear Sirs:* In the June number of your valuable journal there is an article headed "Suspension of the New York College of Dentistry," in which there appears the following assertion: "The difficulties that have for some time existed between the Faculty and Trustees of the institution have resulted in its dissolution." Where you obtained this information, unless from the article quoted from the *American Journal of Dental Science*, it is hard to conceive, and I take immediate occasion to give you the facts in the case, and hope, through your extreme kindness and your good feeling toward the New York College of Dentistry and those connected with it, you will publish the following:

It is true the College is suspended for the time being. It is not true that any difficulties have ever existed between the Faculty and Trustees. It is true that difficulties have existed between the Board of Trustees and the Faculty on the one hand, and the former Dean on the other. It is not true that the College is dissolved or the charter taken from the Trustees, as stated in the article quoted by you. Since the spring of 1868, the date of the last election of the late Dean, it seems to have been his intention to break up the institution, if by any possible means he could accomplish it. It has come to the knowledge of the Trustees that, on several occasions during the year, he has said that the session of 1868 an l 1869 would be the last that the College would hold. This spring, when the Faculty were to be elected, the Alumni of the College drew up a set of resolutions (signed by two-thirds of all the Alumni) setting forth the extreme unpopularity of the late Dean, and praying the Board of Trustees, if they desired the success of the College, to not re-elect him to a professorship. In addition to this the majority of the Faculty refused to serve with him another year. Under the existing circumstances the Board of Trustees did not deem it admissible to re-elect him, and appointed an Auditing Committee to investigate the accounts of the College to date. Having been guilty of gross dereliction of duty as Dean, he received a severe reprimand from several of the members of the Board of Trustees. At this time (April, 1869) Norman W. Kingsley presented a complaint and affidavit (he being the sole complainant) to the Attorney General of the State, paid the required fees and gave the necessary bonds to indemnify the State against any costs in the suit, and the suit was commenced in the name of the people of the State of New York. The charges made against the Board of Trustees for violation of charter have no foundation whatever, and the case was looked upon by every member of the Board as a trivial matter, and they treated it accordingly. A motion for a preliminary injunction on the proceedings of the College was made before Judge Cardoza April 22, and to the utter astonishment of every one. A decision was rendered granting it.

The case has since been re-argued before the same Judge, when the affidavits of all the members of the Board of Trustees, (in the city) except one, were presented denying the charges. The College was represented by John H. Anthon, Esq., (no decision has yet been rendered on the argument) now some five weeks.

The foregoing is a plain statement of the facts relating to the present temporary suspension of the "New York College of Dentistry." At present the Board of Trustees is full and the Faculty complete, with one exception, but under existing circumstances we are prevented from issuing our usual annual announcement, or transacting any other business. The President, Dr. Stephen A. Main, sustained by every other member of the Board of Trustees, (except the complainant) and the entire Faculty, is determined to defend the College against this malicious attack, and feels confident of success.

In corroboration of the above I refer you to the President of the College, Dr. S. A. Main, 23 W. Twenty-third Street; the Vice President, Dr. Wm. H. Allen, 18 W. Eleventh Street; the Treasurer, Dr. John Allen, 22 Bond Street, and the Secretary, M. Mc. N. Walsh, Esq., 67 Nassau Street. Also, to the following members of the Faculty: F. D. Weisse, M. D., Rex W. Stein, M. D., and F. LeRoy Satterlee, M. .D Respectfully yours,

FRANK ABBOTT,
Dean of the Faculty, N. Y. C. D.

